# Navigating blinding challenges in complex intervention trials: insights from a UK researcher survey

**DOI:** 10.1186/s13063-025-09223-9

**Published:** 2025-11-12

**Authors:** Abdullah Yonis, Sarah Gerard Dean, Fiona C. Warren, Rod S. Taylor, William Levack, Jean Hay-Smith

**Affiliations:** 1 Department of Health and Community Sciences, Medical School, University of Exeter, Exeter, UK; 2 School of Health and Wellbeing, University of Glasgow, Glasgow, Scotland, UK; 3 Rehabilitation Teaching & Research Unit, Department of Medicine, University of Otago, Wellington, New Zealand

**Keywords:** Blinding, Bias, Complex intervention, Randomised controlled trials, Survey

## Abstract

**Background:**

One major methodological challenge faced by researchers when conducting randomised controlled trials (RCTs) of complex interventions is blinding. Given the nature of complex interventions, trial participants and intervention providers often cannot be blinded to the intervention. This lack of blinding may result in a lower evidence rating for the trial, potentially hindering the implementation of its findings. One option to mitigate this issue is to single blind studies, with the blinding only of outcome assessors to group allocation. Despite the potential advantages, outcome assessment blinding is frequently underutilised. The challenges behind its limited adoption in this field are not clearly understood. This study aimed to explore the attitudes and practices of UK researchers regarding blinding, particularly outcome assessment blinding, in complex intervention RCTs.

**Methods:**

We conducted an online survey targeting researchers affiliated with the UK Clinical Research Collaboration (UKCRC) Clinical Trials Units (CTUs), the National Institute for Health and Care Research (NIHR), Trials Methodology Research Partnership (TMRP) and National Health Service (NHS)-NIHR research units.

**Results:**

A total of 63 researchers participated in the survey. Most were affiliated with UKCRC (49/63, 77%), TMRP (6/63, 8%) or NHS-NIHR research units (8/63, 12%). The majority (57/63, 91%) agreed that complex interventions pose significant challenges to adequate blinding. Concerns about compromised internal validity due to lack of blinding were noted by 45% of respondents (28/63), and 67% (42/63) expressed dissatisfaction with existing quality assessment tools for complex intervention RCTs. Although 66% of respondents (41/63) found outcome assessment blinding often feasible, 52% (33/63) identified limited resources as a primary obstacle. Free-text responses highlighted practical constraints and additional costs associated with blinding. Furthermore, two-thirds of respondents (43/63, 68%) reported a lack of specific recommendations on blinding, which may exacerbate these challenges. Opinions on the influence of the new Medical Research Council (MRC)-NIHR Framework 2021 were mixed. However, most respondents 76% (48/63)) agreed that NIHR plays a role in improving blinding strategies in complex intervention RCTs and 79% (50/63) agreed that a wider stakeholder engagement is necessary to overcome blinding challenges.

**Conclusion:**

This survey offers insights into the views of experienced UK-based researchers on blinding in complex intervention RCTs. Four key findings emerged: (1) the challenges of blinding due to the inherent complexity of these interventions, (2) the essential role of blinding in reducing bias, (3) the variability in the feasibility of outcome assessment blinding due to practical and resource constraints and (4) the positive impact of stakeholder involvement in addressing blinding challenges. However, respondents expressed dissatisfaction with current quality assessment tools and held divergent views on outcome assessment blinding, highlighting the need for more definitive methodological guidance on blinding in complex intervention trials.

## Background

Blinding is an important methodological approach in randomised controlled trials (RCTs) to minimise the risk of bias by keeping one or more groups of people involved in the trial (e.g. participants, care providers, assessors) unaware of participants’ assigned treatment [1, 2] Blinding seeks to reduce performance and ascertainment/detection bias and helps maintain the integrity of study outcomes [3]. Studies have shown that lack of blinding in RCTs increases the risk of bias and leads to an overestimation of treatment effects [4–7]. Furthermore, poor quality of reporting of blinding in these trials limits the ability of reviewers to judge the risk of bias and quality of evidence [8–10].

Complex interventions consist of numerous components, with interactions both between these components and the surrounding environment.

In recent years, trials involving complex interventions have expanded to encompass a diverse range of populations and settings, with multiple components and outcomes within what is described as a ‘dynamic and adaptive system’ [11]. This ‘complexity’ can hinder the feasibility of blinding [12–15].

However, to ensure the scientific quality of these trials and facilitate the reproducibility of their findings, transparent reporting of blinding procedures and their implementation in complex intervention RCTs is essential [16].

While blinding is widely recognised as a methodological challenge across RCTs of all intervention types, the particular multi-component features of complex interventions present substantial barriers for researchers [16–18].

Strategies, including the use of sham procedures or employing blinded outcome assessors, depend on the specific context of the trial [19]. A researcher’s decision to implement blinding is influenced by several intrinsic and extrinsic factors. Intrinsic factors relate to the trial design, such as population characteristics, health condition of interest and the nature of both the intervention and comparison. Extrinsic factors include the field settings, available resources and logistical constraints. Moreover, stakeholder willingness to support and adhere to blinding can significantly impact its implementation.

One of the fundamental challenges of blinding is the inherent nature of complex interventions, which include multiple components, such as behavioural therapies, educational programmes or multi-step treatments that interact with the surrounding context. These multifaceted aspects make it impractical to blind the intervention providers and the trial participants [20]. These interactions between the integral components of the intervention and surrounding environment may also hinder implementing and standardising outcome assessment blinding [12, 14, 21].The choice of control group(s), such as usual care, waitlist controls or active controls, can further complicate blinding because these groups may not be easily or uniformly masked.

It is therefore important to consider who can be blinded and for what purpose within the trial [22, 23]. The purpose, as well as the challenges, of blinding differ depending on which group within the trial is masked. Blinding participants and care providers primarily reduces performance bias by limiting behavioural or intervention-delivery differences between trial arms [20, 23]. Blinding outcome assessors, in contrast, mitigates detection bias by preventing expectations from influencing data collection or interpretation [24, 25], while blinding statisticians or data analysts helps safeguard against analytical bias during data processing and reporting [26, 27]. Recognising these distinctions is essential when judging the adequacy and feasibility of blinding within complex intervention trials, where it may be possible to blind some groups but not others [22, 28].

In this context, the feasibility of blinding refers to the extent to which it can realistically be achieved in a given trial, shaped by ethical, logistical, technical and operational considerations as well as intervention characteristics, trial design, and available resources [20, 29].

Although the concept of feasibility is frequently used, no formal or universally accepted criteria exist for determining when blinding is ‘feasible’. Most empirical assessments, therefore, rely on expert judgement considering the type of intervention, the nature of outcomes and whether assessors could realistically be blinded without interfering with clinical delivery.

In practice, assessor blinding has been achieved in complex intervention RCTs through several strategies, such as (i) independent endpoint adjudication of objective events (e.g. death or hospitalisation) by committees blinded to allocation; (ii) independent assessors of performance tests (e.g. six-minute walk test) who are not involved in intervention delivery; and (iii) central blinded analysis of imaging, Electrocardiogram or standardised rating scales [30, 31].

The intended outcome is a particularly influential determinant of blinding feasibility [28]. Blinding is generally more feasible with objective outcomes because their measurements do not rely on the perception or interpretation of the participants or assessors. In complex intervention RCTs where outcomes are subjective, knowledge of the treatment allocation can significantly affect how outcomes are reported or assessed. Blinding in this case is more challenging, and the outcomes are more susceptible to biases. For subjective outcomes, outcome assessor blinding is a feasible approach to reduce the risk of bias, provided that third-party assessors, who are unaware of the treatment allocation, are used to conduct the assessments [32].

Outcomes measured through patient-reported outcome measures (PROMs) present particular challenges for blinding. PROMs cannot produce blinded data if participants are aware of their treatment allocation [33–35]. PROMs are increasingly used in trials where the patient experience is an essential component of the outcome [36].

While there is a concern that PROMs used to evaluate the effectiveness of an intervention might lead to an overestimation of treatment effects, they may be the only meaningful or feasible outcomes to measure in certain contexts [35, 37]. However, incorporating additional outcome measures and using blinded assessments when feasible may enhance the rigour of a study, particularly for subjective outcomes.

Regulatory and methodological guidance has cautioned against interpreting PROMs in isolation and recommended combining them with complementary, blinded outcomes as noted in guidance from the U.S. Food and Drug Administration (FDA) [38] and in more recent methodological work by Kluzek et al. [35]. Although such secondary outcomes carry less statistical weight, they can still offer an objective anchor for triangulation and increase confidence if findings are concordant or prompt caution if they are not [39, 40].

The value of outcome assessment blinding may vary depending on the objectives of the trial and the nature of the outcomes being measured. Some researchers suggest that blinding is more crucial when outcomes are subjective rather than objective [41]. Nevertheless, there is a consensus that blinding, particularly outcome assessment blinding, is valuable in reducing the risk of bias and improving internal validity, regardless of the type of outcomes or trial objectives [39, 40, 42]. Generally, the more levels of blinding applied, the greater the protection against bias [26].

Despite the recognised value of outcome assessment blinding, its utilisation in complex intervention RCTs, particularly in rehabilitation and similar continues to be suboptimal [20, 22, 25, 26]. For instance, Kahan found that only 26% of complex intervention RCTs stated that outcome assessors were blinded [25]. The reasons for the infrequent utilisation of outcome assessment blinding are not fully known [26]. Potential factors include practical challenges, resource constraints, a lack of awareness or understanding of available blinding methods, and perhaps, a perceived lack of impact [22, 26, 28].

While the feasibility of blinding has been based mainly on expert interpretation rather than established methodological criteria, available evidence shows that blinding remains underutilised in RCTs, even when it could feasibly be implemented [22, 25, 26]. This is illustrated by Ferrante di Ruffano and Deeks [28] who examined 103 complex ‘test-treatment’ RCTs and judged that while only 22% reported assessor blinding, it would have been feasible in about two-thirds (66%) of them [28].

Boutron et al. [20, 22] similarly suggested that outcome-assessor blinding was often achievable even when participant or provider blinding was impractical [20, 22]. Simple methods include the use of inactive or simulated procedures, placebo acupuncture or mock physiotherapy sessions [43–45].

Further practical evidence can be inferred from three Cochrane reviews of RCTs evaluating complex rehabilitation interventions, namely Dibben et al. [46], (coronary heart disease) Long et al. [47] (heart failure) and McCarthy et al. [48] (chronic obstructive pulmonary disease, COPD) demonstrate that despite broadly similar populations, objective outcomes, settings and country income levels, only around one-third of these trials reported blinded outcome assessment (31%, 35% and 28%, respectively). Thus, across 194 RCTs included in these three reviews, approximately two-thirds of trials did not use blinded outcome assessors, even when outcomes amenable to blinded assessment (e.g. six-minute walk, hospitalisation, cardiac images, Electrocardiogram) were measured. This pattern suggests that underuse of blinding reflects trialists’ contextual and design choices rather than inherent lack of feasibility.

This underutilisation may increase the likelihood of biased results and overestimation of treatment effects, particularly in trials with subjective outcomes [49]. In a large meta-epidemiological study, Page et al. [50] reported that blinding was often not performed in trials with subjective outcomes and lack of blinding was associated with 36% larger treatment effects on average (ratio of odds ratios = 0.64, 95% CI 0.43–0.96).

Given these challenges, the clear reporting of outcomes and blinding status of outcome assessors is even more significant in the reporting of complex intervention RCTs. Detailed reporting of outcomes and blinding is crucial for accurate interpretation and improving clinical decision-making. The Consolidated Standards of Reporting Trials (CONSORT) 2010 statement [51] addressed these aspects in items 6 (outcomes) and 11 (blinding). The statement recommends clearer reporting of blinding by specifying the groups blinded and method of blinding [52].

The CONSORT 2010 statement [51] and its 2017 extension for Non-Pharmacological Trials (NPTs) [53] provide guidelines for reporting outcomes and blinding in RCT reports. CONSORT 2010 recommends specifying all primary and secondary outcomes (Item 6), including details on how and when they were measured, as well as providing details about the blinding of participants, outcome assessors and trial personnel (Item 11) [52]. The NPT extension recommended a thorough description of the non-pharmacological intervention (e.g. complex interventions), detailing control groups (Item 6b and 6c) and also addressing the practical challenges of blinding in such trials (Item 11a) [53].

Quality appraisal tools, such as the Grading of Recommendations Assessment, Development and Evaluation (GRADE) approach used by Cochrane reviewers, are crucial for assessing the quality of evidence from complex intervention RCTs and systematic reviews [54]. The GRADE approach may not necessarily lower the quality of evidence solely due to a lack of blinding, but it urges caution in overestimating the effects of unblinded outcomes and recommends careful interpretation of trial results. GRADE does not explicitly account for practical barriers to blinding when assessing the potential impact of blinding.

Cochrane’s risk of bias (RoB) tool has undergone significant revisions since its inception in 2008 [49]. In 2011, the blinding domain was split into two sections: blinding of participants and blinding of study personnel. However, an audit of the use of Cochrane’s RoB tool in reviews published between 2015 and 2016 revealed confusion about when studies without blinding should be considered at higher risk of bias [55]. Cochrane’s RoB tool was updated again in 2019, and renamed RoB 2, to support more consistent, criteria-driven, theory-informed application of the tool [56]. With RoB 2, the assessment scope was expanded to evaluate if any deviations from the intended interventions were likely to have been influenced by the lack of blinding, and how these deviations might have impacted the study outcomes.

One key difference between RoB 2 and its predecessor is that instead of evaluating risk of bias at the level of whole clinical trials, with RoB 2 risk of bias is assessed at the level of each individual outcome reported in a study [56]. This allows for a much more nuanced approach to evaluating risk of bias with, for example, the opportunity to acknowledge that lack of blinding may have more of an impact on one type of outcome than another [56]. However, even with the improved tool, there have been instances where the tool has been misapplied [57]. A recent paper published by Cochrane editorial and methods group emphasises the importance of using the RoB 2 tool correctly to avoid such problems [57]. The authors cautioned against assuming bias solely based on the lack of blinding, which might result in overly harsh or lenient judgments that could undermine the validity of trial findings [57].

Decisions regarding blinding in these trials can have significant implications for the perceived quality of the study. However, when challenges in achieving blinding arise, evidence from unblinded RCTs remains valuable for informing current practice, guideline development and healthcare policies. Therefore, it is essential to leverage every possible strategy to minimise and to improve reporting transparency. The decisions taken by researchers about blinding in complex intervention RCTs thus have important implications for the judgement of the quality of that study. This in turn informs subsequent decisions in clinical practice, guideline development, and healthcare policies [58].

There is ongoing debate among researchers and methodologists about the value, impact and evidence for blinding in non-pharmacological trials including complex interventions [59]. Anand et al. [60] argue that while blinding is often considered the gold standard for reducing bias in RCTs, its implementation is not always favourable. They noted several undesirable consequences of blinding, such as difficulties in recruitment and retention. For example, blinding may discourage participants who wish to be informed precisely about their allocated interventions. Participants who realise they are not receiving the intervention of interest may withdraw from the study, leading to differential loss to follow-up and bias. Anand et al. emphasised that researchers should prioritise generalisability and relevance over strict adherence to methodological conventions such as blinding [60]. Similarly, Skivington et al. highlight the importance of designing complex intervention trials that are reflective of real-world practice and can generate evidence that is applicable in diverse settings [61].

The updated MRC-NIHR Framework (2021) reflects these evolving perspectives by encouraging researchers to explore diverse methodologies and more flexible approaches to develop and evaluate complex interventions [61]. Although the framework recognised that RCTs are the most suitable experimental design for obtaining an unbiased estimate of the treatment effect, the authors argued that the unbiased estimation of effectiveness should not be the only purpose of evaluating a complex intervention [61]. However, the framework does not specifically discuss the methodological challenges associated with blinding in complex intervention RCTs [61].

This paper presents the results of a survey that explores the perspectives of UK researchers on the challenges of blinding in complex intervention RCTs. The survey identifies key issues related to blinding implementation and the practical challenges of outcome assessment blinding in these trials. While our focus is on the challenges of blinding within complex intervention RCTs, achieving and maintaining blinded outcome assessment is a cross-cutting concern across RCTs irrespective of intervention type. The risk of measurement bias when assessors are unblinded is therefore not unique to this domain, and the researcher experiences reported in this survey are likely to be broadly applicable. The aim is to contribute to ongoing discussions about improving blinding practices, enhancing the reporting of blinding methods, and ultimately strengthening the methodological rigor and validity of complex intervention RCTs.

### Aim

The objective of this study is to explore the attitudes, experiences and practice of UK researchers around blinding, and in particular outcome assessment blinding, in complex intervention RCTs.

## Methods

A cross-sectional online survey was developed in line with established recommendations and practices [62, 63]. We adopted the recommendations of Burford [63] and Eysenbach [64] to guide the survey design, data collection, analysis and reporting.

### Survey design

We developed a customised questionnaire consisting of 30 questions, including multiple-choice, open-ended, and Likert scale questions, with free text boxes for additional comments where appropriate. The survey was structured to present clear questions in a consistent order to each participant. To facilitate ease of use, participants could start, save their progress and return later to complete the survey. Except for an optional free-text field in Question 30, all questions were mandatory to prevent incomplete submissions and reduce missing data. The survey did not use response logic, such as skip or branching logic, to maintain a consistent sequence of questions and enhance data analysis coherence.

### Survey content

After confirming the participant’s willingness to participate, the questions were divided into four sections. The first section included multiple-choice questions about respondents’ backgrounds, job roles and research experience, with closed-list questions allowing for one or more answers. Notably, Question 7 explored the availability of local guidelines on blinding in complex intervention RCTs within respondents’ organisations. This closed-ended question was designed to elicit clear and direct responses regarding the use of general resources or guidelines about blinding (e.g. the Cochrane Handbook).

The second section consisted of 14 questions assessing views on blinding in complex intervention RCTs, using a 5-point Likert scale ranging from strongly agree to strongly disagree. The third section included eight questions on outcome assessment blinding, also using a 5-point Likert scale. The fourth section provided a free-text box for additional comments related to the survey topic*.*

The questionnaire was piloted in two rounds with respondents comparable to the targeted population. Further refinement was achieved through feedback from senior researchers at the Universities of Exeter and Glasgow. A copy of the survey questionnaire is included in the supplementary documents (File 6)*.*

### Survey population

The survey was open to all researchers affiliated with the UK Clinical Research Collaboration (UKCRC) Clinical Trials Units (CTUs) and the Medical Research Council-National Institute of Health Research (MRC-NIHR) Trials Methodology Research Partnership (TMRP). UKCRC’s network of 53 CTUs promotes best practice for delivering high-quality clinical trials across the UK [65, 66]. Some UKCRC researchers were also affiliated with the NIHR Clinical Research Network (CRN) [67], a network of NIHR-funded research units based in National Health Service (NHS) hospitals (referred to as NHS-NIHR research units throughout this paper). Researchers and methodologists involved in complex intervention RCTs in the UK receive significant support from the MRC and the NIHR through partnerships such as the MRC-NIHR TMRP and the UKCRC [66, 68].

### Invitation and participation

The invitation to participate in the survey was extended to researchers with an interest in complex intervention trials. There were no restrictions on participant roles (e.g., clinical investigators, trial managers, statisticians and directors) to gather a broad range of views and insights from individuals involved in or influencing the blinding process. For example, trial managers could provide operational insights about blinding challenges, directors could provide strategic perspectives and statisticians could provide statistical insights.

### Determining the sample size

Given the specific subject area of the survey and the diverse backgrounds of researchers involved in complex intervention trials, setting a precise target sample size was challenging. Although there have been no previous UKCRC surveys specifically focused on researchers’ views regarding blinding, other surveys involving UKCRC registered CTU members conducted between 2014 and 2020 included 30 to 46 respondents [65, 69–74]. Therefore, an achievable target for this survey was set at 50–100 respondents. This target for number of respondents was driven by the decision to include a larger pool of potential respondents than previous surveys, through invitation to UKCRC CTU staff in a range of roles, and respondents from TMRP.

### Survey procedures

Invitations to potential respondents were sent by email to all members on the email list of the 53 UKCRC registered CTUs and the TMRP executive team, either through the administrative team of UKCRC or the TMRP coordinator. The email included a brief explanation of the survey with a link to the participant information Sheet. Informed consent was obtained by completing an online consent form. The survey was hosted on an online platform (Forms/Microsoft 365, 2019). Fully anonymised settings were applied to ensure confidentiality and prevent multiple enrolments. To achieve this, participants were required to sign in using their organisational accounts, which helped confirm that each completion was a unique contribution and limit any duplicate responses.

The survey was available for 6 weeks, from 1st February to 14th March 2022, with reminder emails sent 4 weeks after the start date. All responses were collected in a spreadsheet (Microsoft Excel 2020) and stored on a secure drive. The survey received ethical approval from the College of Medicine and Health Research Ethics Committee at the University of Exeter (Reference: Mar21/B/249).

### Survey analysis

Quantitative responses were analysed using Stata (version 17, 2021) to generate descriptive statistics, such as frequencies and percentages, providing a clear summary of respondent characteristics and survey responses. The first section consisted of multiple-choice questions regarding respondents’ backgrounds and was analysed to provide an overview of participant demographics and professional backgrounds.

Sections two and three of the survey employed a five-point Likert scale to capture participants’ attitudes and perceptions regarding blinding in complex intervention RCTs and outcome assessment blinding. For this analysis, responses of ‘strongly agree’ and ‘agree’ were combined into a single ‘agree’ category and ‘strongly disagree’ and ‘disagree’ were combined into a single ‘disagree’ category. This approach is commonly used to simplify interpretation and draw general trends in agreement or disagreement [75, 76].

### Thematic analysis of free-text responses

Free-text comments were invited to explore researchers’ experiences in various areas of interest, such as barriers to outcome assessment blinding. A thematic analysis was conducted to identify and analyse patterns within these data [40, 77]. The free-text responses were analysed using an inductive coding approach, which allowed themes to emerge from the data. Initially, responses were read thoroughly to understand the content and context. Significant phrases were then assigned short codes; these codes were then grouped into broader themes and refined iteratively.

## Results

A total of 63 responses were received from participants between 1st February and 14th March 2022. The majority of questions were answered by all 63 participants, except for questions 12 and 16, which had 59 responses due to an unforeseen technical issue. Free-text comments were provided by 17 participants, with full details available in Table 5 (see supplementary online file 3). The distribution of responses to questions 2 through 6, which cover participants’ backgrounds, is described in Tables 1, 2 and 3 in the text. Responses regarding the availability of specific guidelines about blinding in complex intervention RCTs (question 7) are presented in Table 4. Responses to questions 8 to 21, concerning blinding practices and challenges, are detailed in Table 6 (see supplementary online file 4). Table 7 provides the details of responses to questions 22 through 29, addressing outcome assessment blinding and related issues (see supplementary online file 5).

**Table 1:** Organisational affiliation of respondents

Respondents’ affiliation	Number of respondents (%) (Total number = 63)
UKCRC CTU	49 (78)
NHS-NIHR funded research units	8 (13)
TMRP executive group	6 (10)
Other healthcare institution	2 (3)

*Some respondents had more than one affiliation

**Table 2:** Respondent job roles

Respondent job role	Number of respondents (%) (*N* = 63)
Statistician	20 (32)
Director or Senior Manager	9 (14)
Research Fellow/Research Associate	7 (11)
Senior Researcher	6 (10)
Trial Coordinator/Manager	6 (10)
Clinician or Healthcare Practitioner	2 (3)
Professor	2 (3)
Public Health Researcher	1 (2)
Other Roles (including Quality Assurance, Scientist)	7 (11)

**Table 3:** Nature of respondent involvement in complex intervention RCTs

a. **Respondent involvement in complex interventions RCTs**	
Involvement type*	Number of respondents (%) (*N* = 63)
Experience in trial design	43 (68)
Authored or published	38 (60)
Trials analysis	37 (59)
Investigator	32 (51)
Leading RCTs	28 (44)
Management of trials	26 (41)
Oversighting trials	2 (3)
Quality Assurance	1 (2)
*Multiple responses were possible	
b. **Respondent length of experience in complex intervention trials**	
Years of experience	Number of respondents (%) (*N* = 63)
0 to 2 years	3 (5)
3 to 5 years	17 (27)
6 to 10 years	12 (19)
More than 10 years	30 (48)
Not stated	1 (2)
c. **Number of RCTs the respondent was involved with in last 10 years**	
Number of RCTs	Number of respondents (%) (*N* = 63)
1 to 5	26 (41)
6 to 10	26 (41)
More than 10	18 (29)
Overseeing RCTs	17 (27)
None	1 (2)

**Table 4:** Availability of specific guidelines about blinding in complex intervention RCTs

*Q: Does your organisation have a policy or recommendations that highlight the value of blinding in complex intervention RCTs?*	Number of respondents (%) (*N* = 63)
Yes	15 (24)
I do not know	5 (8)
No, not at all	21 (33)
No, but there is a general reference (resource)	21 (33)
No, but under development	1 (2)

### Participant characteristics

As anticipated, most respondents were affiliated with UKCRC (49/63, 77%), while some were affiliated with the TMRP Executive Group (6/63, 8%) and NHS-NIHR research units (8/63, 12%) (Table 1).

The respondents held a range of roles, including directors, senior managers, clinical practitioners and statisticians. The survey respondents were an experienced cohort, with 59/63 (94%) having more than 3 years’ experience in complex intervention RCTs, and 30/63 (48%) having more than 10 years’ experience. Additionally, over half (35/63, 56%) had experience in six or more RCTs.

### Challenges of blinding in complex intervention RCTs

A strong majority (57/63, 91%) acknowledged that the nature of complex interventions creates significant challenges for blinding. However, when respondents were specifically asked about blinding trial participants and/or treatment providers in complex intervention RCTs, only 38/63 (60%) reported this was challenging and often impractical. Furthermore, 28/63 (45%) of participants believed the internal validity of these trials had frequently been compromised due to the absence of blinding; 27/63 (44%) neither agreed nor disagreed with this belief, and only 7/63 (11%) disagreed.

### Guidelines about blinding in complex intervention RCTs

Approximately a quarter (15/63, 24%) of respondents reported that their organisations had specific recommendations regarding blinding in complex intervention RCTs, while 42/63 (68%) reported a lack of recommendations within their organisations. Of those who stated their organisations had no local recommendations, half (21/43, 49%) reported the presence of general guidelines (e.g. CONSORT [51], Cochrane guidance [78] and the MRC–NIHR Framework [79]) (Table 4).

### Impact of the MRC-NIHR framework (2021)

The recent MRC-NIHR Framework (2021) [61] was published about 6 months before the survey was conducted. The framework for developing and evaluating complex interventions provides structured principles for intervention development, evaluation and implementation. Its remit extends beyond focusing solely on unbiased estimates of efficacy to encompass process, implementation and contextual factors. These priorities differ from those of methodological guidance such as the Cochrane Handbook, which focuses more directly on trial conduct and risk of bias. By contrast, CONSORT guidance addresses transparent reporting of trial design, conduct and findings. Our survey questions therefore aimed to explore whether this new emphasis in the NIHR Framework might influence researchers’ attitudes towards blinding in complex intervention trials.

Around half of respondents selected the neutral option (i.e. neither agreeing nor disagreeing) when asked whether the NIHR Framework (2021), by shifting emphasis from obtaining unbiased estimates of efficacy towards a broader evaluation remit, would reduce interest in blinding. This included neutral responses to two related questions in this context—30/63 (48%) for the stakeholder-focused item (Q15) and 25/63 (40%) for the researcher-focused item (Q16). Around half of respondents selected the neutral option (i.e. neither agreeing nor disagreeing) when asked whether the NIHR Framework (2021), by shifting emphasis from obtaining unbiased estimates of efficacy towards a broader evaluation remit, would reduce stakeholder interest in blinding (30/63, 48%) or researcher interest in pursuing blinding (25/63, 40%) which may reflect uncertainty about how the Framework will affect practice. Stakeholders, in this context, refer to funding agencies (e.g. NIHR, MRC), healthcare providers, regulatory bodies, patient advocacy groups and policymakers, all of whom have a vested interest in the conduct and outcomes of complex intervention trials. About a quarter of respondents believed the Framework would have a negative impact on stakeholders’ (19/63, 30%) and researchers’ (17/63, 27%) interest in blinding of complex intervention RCTs. In contrast, similar proportions of respondents disagreed, stating that the Framework would not negatively affect blinding practices for either stakeholders (14/63, 22%) or researchers (17/63, 27%).

### Views about outcome assessment blinding in complex intervention RCTs

Two-thirds (42/63, 66%) of respondents agreed that outcome assessment blinding is a reasonable approach to consider in the design of complex intervention RCTs. More than three-quarters (49/63, 78%) agreed that outcome assessment blinding reduces ascertainment bias, and over half (37/63, 59%) agreed that it could enhance the internal validity of a trial. Furthermore, over half (38/63, 61%) did not think that making outcome assessment blinding an essential requirement in complex intervention RCTs would reduce the feasibility of performing these trials. Half (33/63, 52%) agreed that limited resources would be the main obstacle to meeting the requirements of outcome assessment blinding in complex intervention RCTs*.* (see Table 6 and Table 7 [File 4 and File 5] in the supplementary documents). Additionally, respondents also called for quality assessment tools to be better adapted to the unique challenges of blinding in complex intervention trials. Several respondents expressed concerns that existing quality assessment frameworks do not sufficiently account for the specific complexities of blinding in these trials, particularly when assessing subjective outcomes.

### Free-text comments

Seventeen respondents (27%) provided free-text comments related to the survey topics. The comments fell into seven key themes related to challenges and potential approaches to blinding in complex intervention RCTs. Each theme included one or more quotes in the respondents’ own words, which could either come from the same respondent or from different respondents (see Table 5 in the supplementary online file 3 for more details).

Respondents viewed decisions about implementing blinding as primarily the responsibility of the chief investigator and the trial team. Respondents also believed that the trial team ought to assess the feasibility of blinding and consider how to mitigate the risk of bias when blinding cannot be achieved.

There was agreement on the value of outcome assessment blinding, which echoed the positive responses to outcome assessment blinding in the Likert scale questions of the survey. While respondents acknowledged the advantages of blinding, they were also concerned that it required additional resources and poses practical constraints. Balancing methodological rigor with practical constraints was a common concern among the respondents. The blinding of statisticians was a popular theme in the comments as one-third of the respondents (21/63, 33%) were statisticians. There was recognition that it was often impossible to blind statisticians due to technical issues or resource constraints. Others noted the challenge of maintaining statistician blinding throughout the trial duration, particularly when there was only one statistician at a CTU. An outline of the key themes derived from the respondents' free-text comments, along with a selection of quotes, (see Table 5 in the supplementary online file 3 for more details).

## Discussion

### Summary of results

In this survey, an experienced cohort of UK researchers underlined the importance of blinding in complex intervention RCTs, acknowledging its role in reducing bias and enhancing trial quality. While there was general agreement on the value of blinding, respondents had mixed views on its practical implementation in specific contexts. They recognised that outcome assessment blinding helps mitigate detection bias. The respondents saw outcome assessment blinding as going some way towards addressing the overall risk of bias and improving the accuracy of estimating intervention effects.

Nevertheless, they reiterated that inherent design challenges and resource limitations are major barriers to blinding. For instance, outcome assessment blinding is impractical in trials that rely on PROMs. Where outcome assessment blinding in complex intervention RCTs is possible, respondents identified resources and logistical limitations as substantial practical barriers to its implementation.

They emphasized the need for greater support from stakeholders and supporting bodies, such as funding agencies (NIHR, MRC), to address these challenges. Respondents also called for quality assessment tools to be better adapted to the unique challenges of blinding in complex intervention trials.

### Relating our findings to current knowledge

Respondents’ views on the importance of blinding and the difficulties inherent to complex intervention RCTs align with well-established understandings in the methodological literature [5, 20, 28, 53, 80–83]. Our findings indicate that respondents generally support outcome assessment blinding where feasible. This support is encouraging, particularly given that recent literature has highlighted the underutilisation of outcome assessment blinding [84]. For example, a methodological review of 103 open trials found that only 22% implemented outcome assessment blinding, compared to 66% where it was feasible [28]. This support echoes recommendations in Cochrane publications and other literature [25, 32, 80].

A Cochrane review by Fransen et al. [84] on exercise for osteoarthritis of the knee reported several challenges in blinding that relate to the challenges identified in our survey. Out of the 54 trials included in the review, only four trials (7.4%) reported blinding of participants, and slightly more than half (57%) reported blinding of outcome assessors. Although 31 trials stated blinding of outcome assessors, most did not describe how this blinding was implemented. Moreover, the reviewers noted that because the primary outcomes were often self-reported measures of pain and physical function, the effectiveness of assessor blinding was potentially compromised. These observations underscore the survey’s sentiment that design and practical barriers, such as the nature of the intervention and reliance on self-reported outcomes, often limit the feasibility of blinding in complex intervention trials.

There is a well-recognised methodological tension between maximising clinical relevance and minimising risk of bias. Even when outcomes are amenable to blinded assessment in a trial, interactions between assessors and participants can inadvertently compromise blinding [25]. Current good practice therefore recommends monitoring and documenting any instances of unblinding during the trial [20, 41]. CONSORT [51, 53] and SPIRIT [85] guidelines similarly highlight the importance of transparent reporting of whether blinding was maintained, how breaches were identified, and what steps were taken to mitigate them. Therefore, to protect data integrity, trials should anticipate this risk by (i) minimising opportunities for unblinding wherever possible, (ii) recording any instances that occur in a systematic way and (iii) specifying in advance how these data will be analysed, such as by conducting additional sensitivity analyses to examine the effect of excluding or adjusting for unblinded assessments [38, 86].

Although, the survey questions and free-text responses did not specifically explore the relationship between the type of outcomes and the feasibility of blinding, the literature suggests that the type of outcomes being measured significantly impacts both the feasibility and importance of blinding [5, 55, 87–90]. For instance, for objective outcomes, such as blood pressure or laboratory test results, the risk of detection bias is inherently lower because these measures are less susceptible to subjective interpretation by outcome assessors [91]. In contrast, subjective outcomes are highly influenced by participants’ perceptions and assessors’ expectations, making blinding more relevant but also more challenging to implement in order to reduce biases [26].

The assessment of blinding success is particularly contentious in complex intervention RCTs. While maintaining blinding is crucial to reduce bias, the process of evaluating its effectiveness remains both infrequent and controversial [26, 50]. Assessments of blinding success have been criticised for several reasons, including the difficulty in distinguishing between actual bias and participants’ guesses based on perceived benefits or side effects [29, 92]. Reflecting these challenges, the most recent CONSORT guidelines no longer advocate for testing the success of blinding, marking a noticeable shift from the previous stance [26, 52].

Outcome assessment blinding is particularly valuable in complex intervention RCTs, where outcome measures are often subjective [41, 93] and the current body of evidence suggests that trials without outcome assessment blinding may overestimate the benefit of the intervention [59]. When blinding is not feasible, such as in trials that rely heavily on PROMs, using validated instruments that require more than a single item response and well-matched control groups may help mitigate bias [35, 94, 95].

Respondents perceived that quality assessment tools (e.g. GRADE) penalise complex intervention RCTs due to the inherent challenges with blinding [96]. Although the survey responses did not provide specific reasons for this perception, several possible explanations can be considered. One possibility is related to the way reviewers apply RoB tools to assess complex intervention RCTs. For instance, inadequate blinding in these trials might be judged with the same stringency as in pharmacological trials, despite the distinctive challenges faced in complex interventions. The context in which complex intervention RCTs operate often makes it difficult to distinguish between acceptable changes that could introduce some bias and actual bias-inducing deviations. Additionally, the subjective nature of assessing these deviations may vary between reviewers, leading to inconsistent evaluations. Furthermore, the standardised algorithmic approach and signalling questions of RoB 2 may not be sufficiently adaptable to consider the feasibility or lack of feasibility of blinding in the context of these trials. This highlights the challenges facing both researchers conducting complex interventions and those developing quality assessment tools. This sentiment echoes calls by Movsisyan et al. to further develop the GRADE methodology specifically for complex intervention trials [97].

### Implications for current trial practice and future trial methods research

Our findings suggest a shift in the discussion about blinding in complex intervention RCTs from a binary ‘blind all or none’ approach to focusing on feasible levels of blinding and assessing whether blinding significantly reduces the risk of bias and is necessary or beneficial. Although this survey highlighted the practical and methodological challenges of blinding in complex intervention RCTs, such challenges are not unique to this trial type. The underlying issues, such as how outcomes are selected and measured, are central to blinding decisions in most RCTs. Our findings can therefore also be understood in the wider context of blinding, where the overarching aim is to minimise bias and strengthen trial validity.

While the Cochrane RoB 2 tool already evaluates how blinding affects bias in reported outcomes, our survey highlights the need for new considerations in situations where traditional blinding is not feasible.

An important implication here is the need to generate more evidence on how the impact of blinding (including outcome assessment blinding) varies across different domains of complex intervention RCTs. Stakeholders should consider ways to encourage the implementation of feasible levels of blinding when other types of blinding, such as blinding participants or intervention providers, are not feasible. However, in trials where blinded outcome assessment is not possible, particularly those relying solely on PROMs, it is crucial to explore the potential implications, including how the evidence can be interpreted and practically implemented in healthcare practice [38, 86]. This approach requires innovative solutions and a nuanced understanding of the context to ensure the continued production of reliable and meaningful evidence from complex intervention RCTs.

The relationship between blinding in the design of complex intervention RCTs and the success of funding applications is another aspect to investigate. While blinding is at the core of methodological rigor in RCTs and methodological rigor is fundamental to the success of a research proposal in a competitive funding application process [98], only a minority of our respondents linked these two ideas. Additionally, lack of blinding in studies was not seen as a major problem for the uptake of evidence into practice. Further research is needed to understand the impact of blinding and how it is appraised on the uptake and implementation of complex intervention RCTs in clinical practice. This could include qualitative research exploring decision-making about blinding when designing complex intervention RCTs and document analysis of resources used by CTUs to inform decision-making.

Around half of respondents neither agreed nor disagreed with statements suggesting that the NIHR Framework (2021) [61], by shifting emphasis from obtaining unbiased estimates of efficacy towards a broader systems-based evaluation, might reduce stakeholder or researcher interest in blinding. Such neutrality may have several interpretations: it could reflect uncertainty about the Framework’s practical implications, limited familiarity with a relatively new document or a cautious reluctance to judge its applicability to operational blinding challenges. It may also indicate that respondents viewed blinding as a core methodological principle, not necessarily diminished by the Framework’s wider evaluative remit.

Overcoming the barriers to implementing outcome assessment blinding requires engagement with national research bodies to address researchers’ concerns about training and resources for guidance related to blinding in complex intervention RCTs. Tackling the reported gaps in local and national guidance regarding blinding in complex intervention RCTs is also a priority, complementing the most recent MRC-NIHR Framework (2021) recommendations on overcoming these barriers.

### Strengths and limitations

With 63 respondents, we achieved our target of 50–100, surpassing the 40-respondent average of previous UKCRC surveys. One limitation is the potential for self-selection bias associated with voluntary online surveys; however, our survey population included a more diverse mix of roles, such as statisticians, trial managers and directors, compared with previous UKCRC surveys. Another limitation is the difficulty in capturing perspectives on a broad topic through multiple-choice questions. To help mitigate this issue, we provided a free-text option for participants to add comments.

## Conclusion

This survey provides an important contribution to the ongoing methodological discussion around blinding practices in complex intervention RCTs. Our findings highlight researchers’ commitment to enhancing research credibility, recognising the importance of outcome assessment blinding and acknowledging the practical challenges and resource constraints associated with its implementation. Survey respondents also expressed dissatisfaction with current quality assessment tools. There is a need for continued stakeholder engagement on the challenges of blinding in complex intervention RCTs and further methodological research on the value of outcome assessment blinding, to support production of higher quality evidence through better blinding practices and transparent reporting.

### Declaration sections

All relevant survey data, including the questionnaire, responses and the distribution of responses, have been provided in the main manuscript and additional files.

The authors are committed to promoting open science and encouraging the use of this data for academic and research purposes, in alignment with ethical research standards. Researchers interested in accessing further data may contact the authors.

## Data Availability

‘In accordance with the University of Exeter’s Rights Retention Policy, please note that: For the purpose of open access, the author has applied a Creative Commons Attribution (CC BY) licence to any Author Accepted Manuscript version arising from this submission.

## Abbreviations

CONSORT: Consolidated Standards of Reporting Trials
COPD: Chronic obstructive pulmonary disease
CTU: Clinical trials unit
ECG: Electrocardiogram
FDA: U.S. Food and Drug Administration
EMA: European Medicines Agency
GRADE: Grading of Recommendations, Assessment, Development, and Evaluation
MRC: Medical Research Council
NIHR: National Institute for Health and Care Research
NHS: National Health Service
PRO: Patient-reported outcome
PROMs: Patient-Reported Outcome Measures
RCT: Randomised controlled trial
RoB 1: Risk of Bias 1
RoB 2: Risk of Bias 2
SOPs: Standard operating procedures
SPIRIT: Standard Protocol Items: Recommendations for Interventional Trials
TMRP: Trials Methodology Research Partnership
UKCRC: United Kingdom Clinical Research Collaboration
